# Bisphenol A Interferes with Redox Balance and the Nrf2 Signaling Pathway in *Xenopus tropicalis* during Embryonic Development

**DOI:** 10.3390/ani12070937

**Published:** 2022-04-06

**Authors:** Hongjun Chen, Keke Zhong, Yongpu Zhang, Lei Xie, Peichao Chen

**Affiliations:** 1 College of Life and Environmental Science, Wenzhou University, Wenzhou 325035, China; 194511382326@stu.wzu.edu.cn (H.C.); kekezhong@zju.edu.cn (K.Z.); zhangyp@wzu.edu.cn (Y.Z.); 2 Vincent Center for Reproductive Biology, Department of Obstetrics and Gynecology, Harvard Medical School, Massachusetts General Hospital, Harvard University, Boston, MA 02114, USA

**Keywords:** teratogenesis, oxidative stress, DNA damage, RT-qPCR, antioxidant regulator pathway

## Abstract

**Simple Summary:**

Toxicological studies of the effects of BPA on tropical clawed frog (*Xenopus tropicalis*) early embryos show that temporary exposure to BPA during early embryonic development can result in dramatic teratogenesis, DNA damage, and abnormal gene expression. The overall results of this study provide valuable insights for a more holistic assessment of the environmental risks related to BPA in aquatic ecosystems.

**Abstract:**

Bisphenol A (BPA), an environmental estrogen, is widely used and largely released into the hydrosphere, thus inducing adverse effects in aquatic organisms. Here, *Xenopus tropicalis* was used as an animal model to investigate the oxidative effects of BPA on early embryonic development. BPA exposure prevalently caused development delay and shortened body length. Furthermore, BPA exposure significantly increased the levels of reactive oxygen species (ROS) and DNA damage in embryos. Thus, the details of BPA interference with antioxidant regulatory pathways during frog early embryonic development should be further explored.

## 1. Introduction

Bisphenol A (BPA) is currently a widely used plastic monomer and plasticizer. BPA levels were found to be increasing rapidly in the hydrosphere, especially in developing regions [1]. In addition, BPA can be released from a variety of products, including dental sealants [2], tin cans [3], and food contact items [4]. Moreover, alkalinity, incomplete polymerization, high temperature, and other environmental effects can promote the release of BPA into both small and large waterbodies. In large waterbodies, low levels of BPA are maintained due to dilution. For example, the concentration of BPA measured in the water column of the Yong River is 0.07–6.20 nmol/L [5]. From 2004 to 2016, the concentrations of BPA in the Elbe River were found to range from 0.02 to 6.57 nmol/L [6]. In small waterbodies, the main sources of many kinds of toxins are anthropogenic, such as the diffusion pollution of garbage dumps on rainy days and pollution due to chemical fertilizers and pesticides. However, in small waterbodies, such as small lakes, ponds, streams, springs, or ditches, the levels of the pollutants are usually much higher than in large waterbodies [7,8]. It is easily speculated but rarely reported that there are short-term high concentrations of BPA when it is released into the environment. These small waterbodies provide habitats to many species and are thus critical for maintaining freshwater biodiversity and ecosystem service [9]. Therefore, it is necessary to explore the impact of short-term high concentrations of BPA on organisms present in small waterbodies. 

BPA has been well studied as an endocrine disruptor that is structurally similar to diethylstilbestrol (DES) and interacts with estrogen receptors α and β (ERα and ERβ) [10]. In addition to being an endocrine-disrupting compound, BPA has been reported to have a plurality of molecular targets: estrogen-related receptors, G-coupled protein receptors, pregnane X receptors, androgen receptors, thyroid hormone receptors, glucocorticoid receptors, and PPARγ [11]. BPA can interfere with the endocrine system and adversely affect the reproductive, developmental, immune, and other systems of the organisms with which it comes into contact, inducing, for example, abnormal growth patterns and neurodevelopmental delays in children [12]. Previous studies have reported that exposure of *Xenopus laevis* to BPA caused deformities and failed metamorphosis [13,14,15]. In addition to these diverse effects, growing evidence suggests that the induction of reactive oxygen species (ROS) by BPA may contribute significantly to carcinogenesis [16] and reproductive toxicity [17]. 

Nrf2 is a transcription factor that is activated under oxidative and electrophile conditions, thereby transactivating the expression of antioxidant and cytoprotective genes [18]. In addition to its antioxidant roles, Nrf2 is also involved in cell proliferation and the determination of cell fate [19]. Intriguingly, Nrf2 is a maternal gene, and the mRNA levels of Nrf2 decrease up to stage 12 (the neurula stage) in *Xenopus* [20]. Is there a correlation between teratogenesis and ectopic expression of Nrf2, if any, by BPA exposure in early embryonic stages? We hypothesize that BPA exposure induces ROS, which further interfere with the expression pattern of Nrf2 and the following orchestrated embryonic development.

Amphibian adults are usually found on sidewalks or in low-lying grass but normally spawn in small waterbodies such as temporary static ponds and puddles formed after heavy rains. Due to their unshelled eggs and highly permeable skin, their embryos and tadpoles are susceptible to contamination, especially in these small waterbodies. Therefore, in this study, we utilized the embryos of a model animal, i.e., *X. tropicalis*, to investigate the oxidative effects of BPA during early embryonic development (prior to the neurula stages), as well as any prolonged toxicity and effects during later development stages (tadpole stages). We found that BPA exposure increased ROS levels and interfered with the Nrf2 signaling pathway in embryonic development, suggesting an association with teratogenesis. These results can also serve as a reference for the effects of BPA on human and vertebrate embryonic development.

## 2. Materials and Methods

### 2.1. Exposure and Sampling

*X. tropicalis* (Nigerian) embryos were provided by Xiao Huang, Zhejiang University, and were bred and maintained in our lab. The animal research procedures were conducted in compliance with the guidelines of the Wenzhou University Animal Care and Use Committee. According to the requirements for indoor tropical aquatic breeding, the frogs were maintained in a water circulation system, the temperature was controlled at 28 ± 0.5 °C, and the photoperiod was 12 h light/12 h dark. Thirty pairs of mature male and female frogs were induced to mate by injecting them with 150 U and 200 U of human chorionic gonadotropin (Ningbo Second Hormone Factory, Ningbo, China), respectively, in the dorsal lymph sac, thereby inducing the females to start spawning 4 h later. Instead of dissecting the testis to perform in vitro fertilization (IVF), we allowed them to participate in amplexus. To obtain synchronic embryos, we collected the embryos every 30 min, which differed by a half developmental stage. The embryos were slight dejellied using 2% cysteine (pH 8.0) until they could be separated. Then they were washed with 0.1 × Marc’s modified ringer solution (MMR) immediately to stop the dejelling and cultured in 0.1 × MMR. The developmental stages were assessed according to those previously described [21]. 

Because *X. laevis* did not survive even for 48 h when treated with 100 μmol/L BPA [14], high concentrations and 12 h exposure were set for further exploration of the toxicology effects of BPA. For the BPA exposure experiment, BPA (Sigma-Aldrich, St. Louis, MO, USA) was dissolved in dimethyl sulfoxide (DMSO, Sigma-Aldrich, St. Louis, MO, USA) to 4 mol/L for the stock solution. DMSO may be used as a solvent without complications at a maximum concentration of 0.01% [22]. The stock solution of BPA was diluted in 0.1 × MMR to 25, 50, and 100 μmol/L (0.000625%, 0.00125%, and 0.0025% DMSO, respectively). Each treatment contained three 15 cm glass dishes, and each dish consisted of 200 mL test solutions and 100 randomly selected embryos. Embryos at the four-cell stage were cultured in 0.1 × MMR containing different concentrations of BPA or 0.01% dimethyl sulfoxide (control group) for 12 h at 26 ± 1 °C, followed by another 60 h without BPA. The test solution was changed every 6 h for the first 12 h, and every 12 h thereafter. During the exposure, dead embryos were counted and removed, and abnormal embryos were counted every 12 h. Dead embryos could be recognized by the presence of gray or white patches on the embryo surface. Apparent malformations were recorded based on the Atlas of Malformations [23] and compared with those found in control embryos.

### 2.2. Detection of Levels of Reactive Oxygen Species (ROS)

Reactive oxygen species (ROS) were detected using 2,7-dichlorodihydrofluorescein diacetate (H_2_DCFDA, Sigma-Aldrich, St. Louis, MO, USA) following a standard protocol [24]. H_2_DCFDA was used to determine the hydrogen peroxide (H_2_O_2_) concentration within the embryos [25]. Fifteen normal live embryos were randomly selected from each dish for ROS detection and were pooled into one sample (three mixed samples per treatment group) at time points of 12 and 72 h. The fluorescence intensity of ROS in *X. tropicalis* embryos was detected using a fluorescence microplate reader (Cytation 3M, BioTek, Winooski, VT, USA) with an excitation wavelength of 485 nm and an emission wavelength of 520 nm.

### 2.3. RNA Isolation and Quantitative Reverse Transcription PCR (RT-qPCR)

At the 12 h time point, 15 embryos were randomly selected from each dish and were pooled into one sample (three samples per treatment group) for RNA extraction. Since RNA is easily degraded, embryos need to be frozen. Before freezing, 1g/L MS-222 was added as anesthesia, and the samples were then frozen using liquid nitrogen. Total RNA was extracted from frozen embryos using TRIzol Reagent (Life Technologies, Carlsbad, CA, USA), and the quality of RNA was established using gel electrophoresis and NanoDrop 2000 (ThermoFisher, Waltham, MA, USA). According to the procedure, 1 μg of total RNA was reverse-transcribed to cDNA using oligonucleotide (dT)-tailed primer and Reverse Transcriptase M-MLV (Takara, Shiga, Japan). Before RT-qPCR, gel electrophoresis was used to verify the specificity of the amplified products. In addition, the melting curves were assessed as another method for verification of specificity during the RT-qPCR process. RT-qPCR reactions were performed in triplicate for each sample, using FastStart Universal SYBR Green Master (Roche, Basel, Switzerland) in a CFX-Connect Real-Time System (BIO-RAD, Hercules, CA, USA). Based on the studies of *X. laevis* [26], β-actin was taken as the reference gene, and the gene-specific primer sequences are listed in Table 1. The efficiency of these genes ranges from 94.27% to 102.35%, which corresponds to an amplification efficiency between 90 and 110%. The relative mRNA expression level of each gene was normalized to that of the reference genes and calculated using the 2^−ΔΔCt^ method.

### 2.4. Whole-Mount In Situ Hybridization (WISH)

Nrf2 sequences were retrieved from the NCBI and Xenbase (http://www.xenbase.org, accessed on 9 February 2022) databases. Following PCR amplification, fragments of Nrf2 were cloned into the PCS107 plasmid. The plasmids were then linearized using BamHI and the mMACHINE SP6 Transcription Kit (Thermo Fisher, Waltham, MA, USA) was used to synthesize the DIG-labeled antisense fragment. Three normal live embryos in the control and 25 μmol/L groups were randomly selected from each dish for WISH at stages 19, 32, and 38 (nine samples per treatment group per stage). Then, the embryos were fixed in MEMFA (0.1 mol/L MOPS, 2 mmol/L EGTA, 1 mmol/L MgSO_4_, 3.7% formaldehyde, pH 7.4) for 1–2 h at room temperature and completely dehydrated using different grades of absolute ethanol. WISH was performed according to a previously described procedure [27]. Alkaline phosphatase (AP)-coupled anti-DIG antibody was used to recognize the DIG-labeled probe. Staining was conducted in BM Purple (Roche) at 37 °C. The embryos were observed under a stereomicroscope (SteREO Discovery. V8, Carl Zeiss, Jena, Germany), and the images were taken using a digital camera (AxioCam MRc, Carl Zeiss, Jena, Germany). 

### 2.5. Micronucleus Test

Each treatment was conducted on 30 tadpoles (around Stage 47), and 10 tadpoles were kept per 10 L glass tank filled with 2 L of different BPA concentrations (25, 50, and 100 μmol/L) or 0.01% dimethyl sulfoxide (control group) for 6 h, before being moved to 0.1 × MMR for another 24 h. Three tadpoles per group were randomly selected and euthanized; then, blood was immediately collected from the tadpole heart to prepare a blood smear. Two smears per tadpole, fixed in methanol for 15 min, were then stained with 10% Giemsa for 30 min. Subsequently, the slides were rinsed with deionized water 3–5 times, before air-drying for microscopic examination. The number of cells that contained one or more micronuclei was determined in a total sample of 1000 blood cells per tadpole. Then, the micronucleus rate was calculated, taking the average (MCN‰). 

### 2.6. Statistical Analyses

In this study, descriptive data (mean ± SD) were generated for every dependent variable. The mortality, deformity, and micronucleus rates were compared pairwise by chi-squared testing according to a previous study [28]. Before one-way analysis of variance, Shapiro–Wilk was used to test the distribution normality, and Levene’s test was used to test the homogeneity of variance. The data for *SOD2* and *CAT* were compared after conversion (*SOD2* was converted by the ln function; *CAT* was converted by the sin function). The average difference between control and treatment was evaluated by one-way analysis of variance (ANOVA) and the least significant difference (LSD) post hoc test; a *p*-value of < 0.05 was considered statistically significant. 

## 3. Results

### 3.1. BPA Causes DNA Damage in the Tadpole

**Figure 1:**
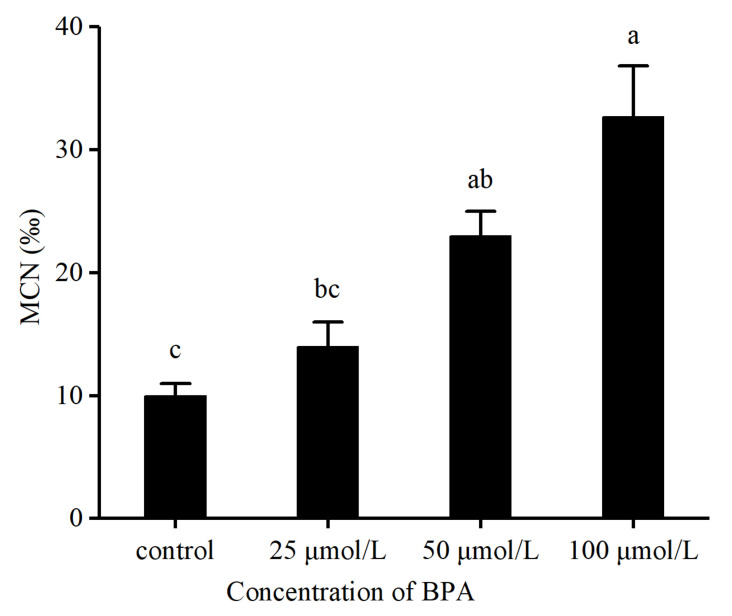
BPA exposure causes later micronuclei in blood cells. The tadpoles were exposed to BPA for 6 h before refreshing the water, and the micronucleus rate was measured again after 24 h. Different letters above the bars indicate significant differences among groups (a > b > c, *p* < 0.05). n = 3/treatment.

Results showed that the micronucleus rates were 10‰ ± 1‰, 14‰ ± 2‰, 23‰ ± 2‰, and 33‰ ± 4.16‰ for the control and 25, 50, and 100 μmol/L BPA exposure groups, respectively (Figure 1). Exposure to 50 and 100 μmol/L BPA significantly increased the micronucleus rate (χ^2^ = 46.94, *p* < 0.05). 

### 3.2. BPA Is Teratogenic to X. tropicalis Embryos

**Figure 2:**
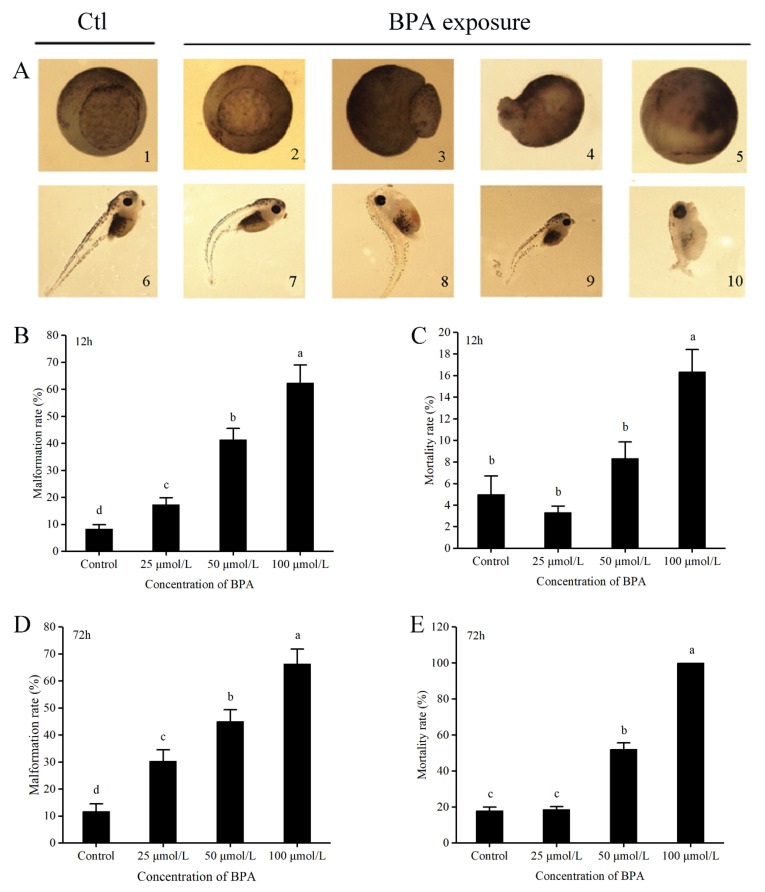
BPA induces malformation during *X. tropicalis* embryonic development. The embryos were exposed to different concentrations of BPA for 12 h; when the BPA was removed, the embryos were at stage 10.5 (early gastrula) ((**A**) 1–5), and the malformation analysis ended at the later tadpole stage 46 ((**A**) 6–10). The malformation rates were statistically analyzed at 12 h (stage 10.5) (**B**) and 72 h (stage 46) (**D**). The mortality rates were statistically analyzed at 12 h (stage 10.5) (**C**) and 72 h (stage 46) (**E**). Different letters above the bars indicate significant differences among groups (a > b > c > d, *p* < 0.05). n = 300/treatment.

All the embryos at the four-cell stage were exposed to different concentrations (25, 50, and 100 μmol/L) of BPA for 12 h. At this endpoint, the embryos arrived at the early gastrula stage (Figure 2(A1)). Treating with BPA was found to result in developmental delays (Figure 2(A2)), abnormal blastopore closure (Figure 2(A3)), and death (Figure 2(A4,A5)). Compared with the control group, all BPA treatment groups showed a significant increase in these factors (χ^2^ = 244.35, *p* < 0.05, Figure 2B). In addition, the mortality rate showed a significant dose-dependent increase (χ^2^ = 39.67, *p* < 0.05, Figure 2C). 

After 12 h of BPA exposure, the surviving embryos were transferred to fresh 0.1× MMR and allowed to develop for another 60 h. They were estimated to have arrived at the late tailbud stage in normal conditions (Figure 2(A6)). The embryos in the BPA treatment groups showed flexure of the tail (Figure 2(A7)), pericardial edema (Figure 2(A8,A10)), hypopigmentation (Figure 2(A7,A8,A10)), and a shorter tail (Figure 2(A10)). The most characteristic malformation was a shortened body length (Figure 2(A7,A10)). The malformation rate at the 72 h time point exhibited the same pattern as that at the 12 h time point (χ^2^ = 203.51, *p* < 0.05, Figure 2D). As shown in Figure 2E, the mortality rates were also significantly increased along with the dose-dependent increase in BPA concentrations (χ^2^ = 539.05, *p* < 0.05, Figure 2E).

### 3.3. BPA Enhances Antioxidant Signaling during Embryonic Development

**Figure 3:**
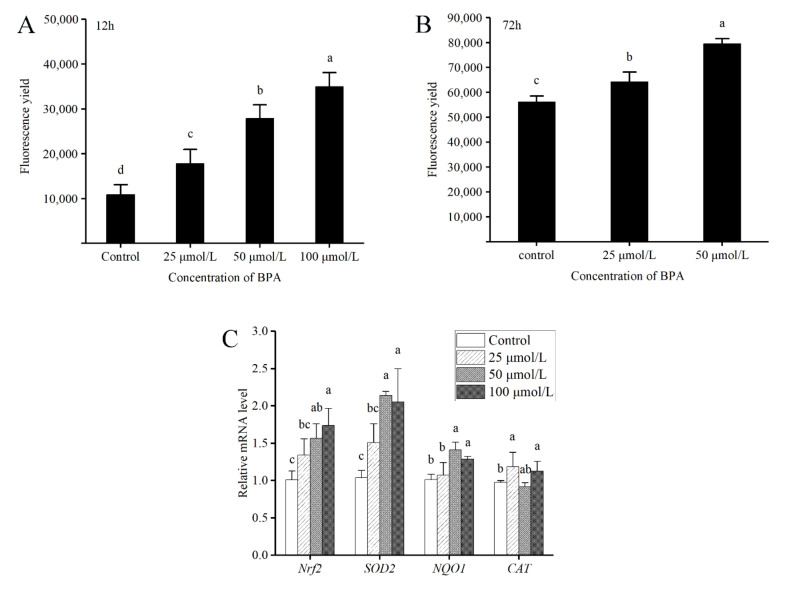
BPA exposure enhances redox signaling during embryonic development. The ROS levels were measured at the time of BPA removal (after exposure for 12 h) (**A**) and at the statistical analysis endpoint (72 h) (**B**). n = 3/treatment. (**C**) The representative genes involved in the redox signaling pathway were determined at the time of BPA removal (12 h). Different letters above the bars indicate significant differences among groups (a > b > c > d, *p* < 0.05). n = 3/treatment.

ROS levels were measured after the termination of BPA exposure (12 h) and after a further 60 h of development (72 h). As shown in Figure 3A, ROS levels in the embryos were significantly increased by BPA (*F*_3,8_ = 41.37, *p* < 0.05) in a dose-dependent manner. After removal of BPA, the ROS levels of the 25 and 50 μmol/L BPA treatment groups also showed a significant increase compared to the control (Figure 3B, *F*_2,6_ = 51.27, *p* < 0.05). 

At 12 h, significant upregulation of Nrf2 and *NQO1* was observed in the 50 and 100 μmol/L BPA groups compared to their levels in the control (Figure 3C, *F*_((Nrf2) (3,8))_ =7.79, *F*_((NQO1) (3,8))_ =8.63, *p* < 0.05). *SOD2* expression was significantly upregulated compared to the control in embryos exposed to 25, 50, and 100 μmol/L BPA (Figure 3C, *F*_3,8_ = 11.57, *p* < 0.05). Compared to the control, the mRNA level of *CAT* was not significantly changed in the 25, 50, and 100 μmol/L BPA groups.

### 3.4. BPA Interferes with the Spatiotemporal Expression Patterns of Nrf2

**Figure 4:**
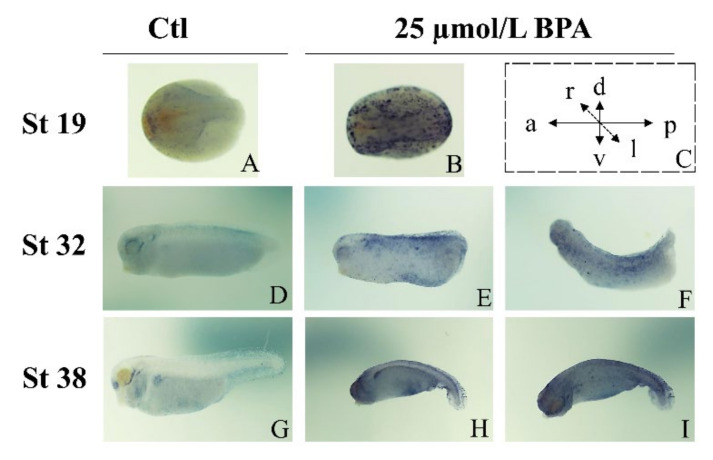
BPA exposure disrupts the spatiotemporal Nrf2 expression patterns during embryonic development, determined using whole-mount in situ hybridizations; representative phenotypes are shown for the control (**A**,**D**,**G**) and treated (**B**,**E**,**F**,**H**,**I**) groups, according to the scheme in (**C**): a, anterior; p, posterior; v, ventral; d, dorsal; r, right; l, left (**C**). n = 9/treatment.

We further examined the spatiotemporal expression patterns of Nrf2 by WISH. During the neurula stages, the mRNA expression levels of Nrf2 in previous BPA-exposed embryos were significantly increased in the neural crest and neural plate, compared to the control embryos (Figure 4A,B). Following exposure to BPA, a significant upregulation of Nrf2 was observed in the tail and spinal cord, with curvature of the tail and trunk observed at stage 32 (Figure 4D–F). Later, at stage 38, Nrf2 was upregulated in the cloaca, tail, and spinal cord following previous BPA exposure (Figure 4G–I). This showed that BPA upregulated the mRNA expression levels of Nrf2 in the malformed parts of the embryos, suggesting that Nrf2 is linked with teratogenesis following BPA exposure during the early embryonic stages.

## 4. Discussion

Embryogenesis is an extremely complex but delicately controlled process. According to the spatiotemporal coordination of cell specification and cell migration, the fertilized egg develops into a shaped individual, which mainly occurs within a short period termed gastrulation [29]. Therefore, even a tiny error at the gastrulation stage will result in obvious adverse outcomes, such as malformation and death. The expression level of Nrf2 decreased from the blastula stage, and reached a minimum at stage 12 [20]. Therefore, BPA treatment of embryos from the four-cell stage to the early gastrulation stage (approximately 12 h in duration) was conducted to investigate whether there is a correlation between the teratogenicity caused by BPA exposure and ectopic Nrf2 expression in early embryos. We aimed to uncover the oxidative effects of BPA during embryogenesis, focusing on discerning the effect of BPA exposure during early embryonic stages. 

In this study, increased BPA exposure increased the deformity and mortality rates of *X. tropicalis* embryos. Several studies have found that exposure to high concentrations of BPA induces rapid and high mortality in *X. laevis*, zebrafish embryos, and *Artemia* nauplii [30,31,32]. However, our results show that the mortality rate of 100 μmol/L BPA treatment was 16.33% ± 2.08%. The different mortality rates can be explained, in part, by the difference in susceptibility between different species. In addition, differences in exposure time points and periods also result in different mortality rates. 

We found that treatment of *X. tropicalis* embryos with BPA resulted in developmental delays (Figure 2(A2)), abnormal blastopore closure (Figure 2(A3)), death (Figure 2(A4,A5)), flexure of the tail (Figure 2(A7)), pericardial edema (Figure 2(A8,A10)), hypopigmentation (Figure 2(A7,A8,A10)), and a shorter tail (Figure 2(A10)). Among these effects, the most typical deformity was a shortened body length (Figure 2(A7,A10)). The shortened body length may reduce the growth and survival rate and predation by natural enemies [33]. In addition, it was recently reported that BPA (1-50 μmol/L) treatment in *X. laevis* caused disrupted cleavage divisions, slowed cytokinesis and cellular dissociation, and resulted in subsequent teratogenesis [34]. The shorter tail and shortened body length observed in our study are consistent with these reported deformities, though we did not observe a curved spinal cord and craniofacial malformations. A relevant study has shown that BPA can cause *X. laevis* eye dysplasia by affecting Notch signaling [35]. BPA exposure significantly interferes with cell specification and cell migration during embryogenesis, though the underlying mechanisms are largely unclear. 

The micronucleus test is a simple, rapid, and effective screening method to detect the damage of the cell genetic material (i.e., chromosome) caused by environmental pollutants [36]. It has been recommended by many countries and international organizations as one of the genetic toxicology methods to detect carcinogens and mutagens [37]. The micronucleus rate in BPA treatment groups was found to increase with the increase in BPA concentration (Figure 1), indicating an increase in DNA damage following BPA treatment. The DNA damage caused by BPA was thought to be caused by the indirect effect of ROS [38].

It is known that short-term exposure to micromolar doses of BPA can increase the level of oxidative stress [38,39], which could lead to lipid, protein, and polysaccharide oxidation and DNA damage, disrupting the process of apoptosis in the process of organ formation [40]. Even after removing exogenous BPA, increased ROS levels can persist [39]. Due to the fragile environment of the embryo, it is extremely vulnerable to damage by ROS and, thus, ROS damage affects embryo development via the impact on physiological and pathological processes in subsequent stages [41]. To avoid oxidative damage, antioxidant systems such as SOD and CAT play an important protective role. SOD can catalase the conversion of superoxide dismutase into oxygen and H_2_O_2_ [42], while CAT can convert H_2_O_2_ into water and oxygen, thereby reducing toxicity [43]. Previous studies have demonstrated that BPA can induce oxidative stress by altering the CAT and SOD activity or the transcription of antioxidant-related genes in mice and rats [16]. In the present study, H_2_O_2_ concentrations and *SOD2* expression levels increased in the embryos after exposure to BPA, which is consistent with the previous study. This might indicate that BPA causes deformities and deaths by inducing oxidative damage in *X. tropicalis* embryos.

Moreover, the Nrf2 pathway also plays a vital role in resisting redox stress [44,45,46]. In addition, it was found that BPA can activate the Nrf2 signaling pathway at high micromolar concentrations (>10 μmol/L) [47]. In the present study, Nrf2 expression was upregulated by 25–100 μmol/L BPA and the expression of *NQO1* and *SOD2* followed the tendency of Nrf2, which is consistent with the previous studies. However, the expression of *CAT* showed a weak relation to Nrf2 in embryos. We speculate that the mechanisms regulating *CAT* in response to BPA exposure are complex.

Nrf2 is known to play an important role in cell fate specification [48] by affecting the Notch signaling pathway [49], which is involved in developmental processes. Therefore, Nrf2 may be a key molecule that affects stem cell renewal and cell fate in embryonic and adult tissues [49,50]. In the present study, BPA-induced Nrf2 overexpression may affect cell differentiation and migration during embryogenesis, suggesting that the overexpression of Nrf2 in the embryo interferes with cell migration, thus preventing the embryonic pores from being completely closed (Figure 2(A2,A3)).

## 5. Conclusions

In this study, we provided evidence that temporal exposure to BPA during early embryonic development stages results in dramatic teratogenesis. Furthermore, following BPA exposure, the expression of the Nrf2, *NQO1*, and *SOD2* genes was upregulated in response to 25-100 μmol/L BPA treatments, suggesting that the Nrf2 signaling pathway and redox balance was disrupted, which may be associated with the teratogenic effects of BPA. It is suggested that organisms living in small waterbodies might be threatened by BPA. Therefore, more research should be carried out to evaluate the potential hazards of BPA to human beings and ecosystems.

## Figures and Tables

**Table 1:** Primer sequences.

Genes	Forward Primer (5′–3′)	Reverse Primer (5′–3′)
*NQO1*	TTTTGTCCTTTACTACGGGG	CACTCTTCTGCTATCTCTGT
Nrf2	CACCAGAGACGAGCAAAGAG	CTTTGTCTGCTGGAGGGAGT
*SOD2*	GCCAATCAAGACCCTCTACAA	TTTCGGCTACATTCTCCCAGT
*CAT*	ACTGCAGAGCCAAGGTGTTT	TATCGGGGTCCTTCAGGTGT
*β-actin*	GCTGCTTCTTCTTCATCAT	TTGGCATAGAGGTCCTTAC

## Data Availability

Data are available from the authors upon request.
